# Computational Protein Phenotype Characterization of IL10RA Mutations Causative to Early Onset Inflammatory Bowel Disease (IBD)

**DOI:** 10.3389/fgene.2018.00146

**Published:** 2018-04-27

**Authors:** Fahad A. Al-Abbasi, Kaleemuddin Mohammed, Saida Sadath, Babajan Banaganapalli, Khalidah Nasser, Noor A. Shaik

**Affiliations:** ^1^Department of Biochemistry, Faculty of Science, King Abdulaziz University, Jeddah, Saudi Arabia; ^2^Princess Al-Jawhara Al-Brahim Center of Excellence in Research of Hereditary Disorders, King Abdulaziz University, Jeddah, Saudi Arabia; ^3^Department of Genetic Medicine, Faculty of Science, King Abdulaziz University, Jeddah, Saudi Arabia; ^4^Department of Medical Laboratory Technology, Faculty of Applied Medical Sciences, King Abdulaziz University, Jeddah, Saudi Arabia

**Keywords:** *In-silico* analysis, IL10RA gene, pathogenic mutations, inflammatory bowel disease (IBD), molecular docking

## Abstract

The deleterious amino acid substitution mutations in IL-10 receptor alpha gene are most frequently reported in several autoimmune diseases including early onset-inflammatory bowel disease (IBD). Despite the important role of IL-10 RA in maintaining immune homeostasis, the specific structural and functional implications of these mutations on protein phenotype, stability, ligand binding and post translational characteristics is not well explored. Therefore, this study performed the multidimensional computational analysis of IL10RA missense variations causative to pediatric or early onset inflammatory bowel disease (<5 years of age). Our computational algorithmic screening identified the deleterious nature of p. W45G, p. Y57C, p. W69G, p.T84I, p.Y91C, p.R101W, p.R117C, and p.R117H, IBD causative IL10-RA mutations. The sensitivity and specificity analysis of different computational methods showed that CADD outperform SIFT, PolyPhen 2.0, FATHMM, LRT, MetaLR, MetaSVM, PROVEAN and Condel in predicting the pathogenicity of IL10RA mutations. Our three-dimensional protein modeling assays showed that the point mutations cause major drifts in the structural plasticity of IL10 RA molecule and negatively influence its stability. Findings from molecular docking analysis have shown that these point mutations decrease the binding affinity of IL10RA toward IL10 and may likely to disturb the IL10 signaling pathway. This study provides an easy frame work for phenotypic characterization of mutant IL10RA molecule in terms of structure, flexibility and stability aspects. Our approach may also add a new dimension to conventional functional biology assays in quickly studying IL10 RA mutations and also for designing and developing inhibitors for mutant IL10RA molecule.

## Introduction

IL10RA (Interleukin-10 receptor alpha) essentially constitutes to Interleukin-10 receptor hetero tetramer, that belongs to class II cytokine or the IFNR-like receptors (Ho et al., 1993; Tan et al., 1993; Liu et al., 1994). The two α sub-units of IL10RA interacts (Kd ~35–200 pM) with IL10 (Josephson et al., 2001) and forms a high affinity IL10-IL10RA complex. This intermediate complex forms a low affinity interaction with IL10RB, subsequently forming an active signaling complex that activates Janus Kinase 1 and Tyrosine Kinase 2 (Finbloom and Winestock, 1995), leading to nuclear translocation of Signal Transducer and Activator of Transcription 3, activation of downstream target gene transcription of anti-inflammatory effectors (Stahl et al., 1995). IL10 is a critical cytokine molecule that modulates the intestinal mucosal homeostasis. This is supported by the description that, laboratory mice deficient in IL10 prone to develop spontaneous enterocolitis (Shouval et al., 2014). In concordance to these findings mice deficient in IL 10 receptor is also seen to develop spontaneous enterocolitis (Spencer et al., 1998). In humans, both IL10 and its receptors, together contribute in controlling the intestinal mucosal immune responses. Nucleotide sequence alterations in IL10 are linked with the risk of developing inflammatory bowel disease (IBD), as reported in genome wide association studies (Mesbah-Uddin et al., 2015; Huang et al., 2017). A severe early onset IBD, which occurs in children (<5 years) is reported to occur in individuals with mutations in IL10 or its receptors (Zhu et al., 2017). Mutations in coding region of IL10 or IL10 receptor alpha or beta genes leads to defective IL10 signaling and disturb the anti-inflammatory responses in the gastro intestinal tract. The known early onset IBD causative mutations of IL10RA gene includes W45G, Y57C, W69R, T84I, Y91C, R101W, R117C, R117H, L125R, G141R, I169T, I224V, R262C, R412W, and R412Q. However, their phenotypic characterization in terms of structural plasticity, stability and IL10 binding characteristics aspects is not yet performed.

The characterization of missense mutations through conventional laboratory assays is expensive, time taking and laborious. In this regard, the recent studies indicate that computational approaches can successfully explore the deleterious effects of different mutant versions of protein molecules (Banaganapalli et al., 2016, 2017). Therefore, we aimed to understand the molecular reasons behind detrimental effects of mutant IL10RA molecules, through comprehensive computational assays covering pathogenicity predictions, structural characteristics determination and analysis of ligand binding characteristics. As an initial step, we recorded all the deleterious mutations in IL10RA from the publicly available databases and shortlisted them based on algorithmic predictions. In the next step, structural analysis was carried out, to investigate how one amino acid change can create an undulate effect throughout the protein structure and eventually affect the protein function. Based on the crystal structure (1Y6K; 22-235 residues) of native IL10RA, we generated 13 full-length (1-578 residues) template-based models (one wild-type and 12 mutant models), by using Swiss Model-Expasy and I-TASSER web servers. Consequently protein-protein interaction analysis and molecular docking of selected mutant (localized to the extracellular region) and native models of IL10RA with its ligand IL10 (highly interacting partner) was performed to check the ligand binding characteristics. Furthermore, *in-silico* functional analysis was conducted to identify the mutations which undergoes post-translational modifications (PTM) of IL10RA protein. By analyzing the early-onset IBD causative mutations of IL10RA gene, this study provides the computational in-sight into the structural and functional aspects of IL10RA.

## Methodology

### Data sets

The mutations of IL10RA gene (CCDS Transcript ID: ENST00000227752), their corresponding IDs and aminoacid sequences were obtained from NCBI dbSNP, UniProt, Exome Variant Server, and Ensembl databases. Later IBD causal variants were shortlisted from the pool of IL10RA non-synonymous missense mutations. In addition, recently published research articles and reviews were screened to collect the reported IBD variants. Additionally, mutations reported in the databases like “Web of Science” “PUBMED,” and “Cochrane-Library,” were also incorporated. The keywords used to search the mutations were “Inflammatory bowel disease,” “IBD,” “infantile IBD,” “early onset IBD,” “IL10RA,” “IBD,” “SNPs,” “mutation,” “Polymorphism,” and “Variation.” The data was further enriched by eliminating the redundant mutations collected different data resources.

### Pathogenicity prediction

To predict the deleterious nature of the mutants, we employed dbNSFP at Ensemble VEP (Variant Effect Predictor) (McLaren et al., 2016) which produces prediction scores for different algorithms like SIFT, PolyPhen 2.0, CADD, FATHMM, LRT, MetaLR, MetaSVM, PROVEAN, and Condel. These tools works on diverse principles like sequence homology, structural homology and difference in Gibbs free energy values etc. These tools require mutation related input entries from the user and the results revealed in different forms with specific threshold points, cut-off values and correlation.

SIFT, CADD, FATHMM, and LRT are nucleotide sequence conservation-based algorithms; PolyPhen-2.0 and PROVEAN are structural-homology based approaches while Condel, MetaLR, and MetaSVM runs on integrated algorithm. Most of these tools demand the query in the form of chromosome number and allele change whereas few other require protein sequence with mutation position.

### Concordance analysis

To enhance the prediction accuracy, results from different computational tools were combined and a correlation analysis was performed. An integrated ranking code was developed, as per the following scheme: variants that are predicted deleterious by all the 9 tools were referred as rank-I, whereas, variants that were predicted to be deleterious by 5–8 tools are referred as rank-II and rank-III variants predicted to be deleterious by less than 5 tools. Sensitivity analysis was conducted to assess the heterogeneity of computational tools. Receiver Operating Characteristic (ROC) curve was initially constructed by plotting “sensitivity” and “specificity” of each tool and the curve fitting was performed by DeLong et al. (1988) model with binomial exact confidence interval (95% CI) using MedCalc Statistical Software version 16.8.4 (MedCalc Software bvba, Ostend, Belgium, Schoonjans et al., 1995). 13 benign variants were added to the test set, along with 15 disease causal variants, for accuracy analysis. Area under the curve (AUC), specificity and sensitivity values were obtained by MedCalc, whereas Accuracy and Mathews coefficient correlation (MCC) was calculated by following formula (details are provided in the supplementary document)

$$\begin{array}{l} \text{Accuracy}=\frac{\left(\text{TP}+\text{TN}\right)\;}{\left(\text{TP}+\text{TN}+\text{FP}+\text{FN}\right)} \end{array}$$

$$\begin{array}{l} \text{MCC}=\frac{\left(\text{TP }\times \text{ TN}\right)\;\left(\text{FP }\times \text{ FN}\right)}{\sqrt{\left(\left(\text{TP}+\text{FP}\right)\;\left(\text{TP}+\text{FN}\right)\;\left(\text{TN}+\text{FP}\right)\;\left(\text{TN}+\text{FN}\right)\right)}} \end{array}$$

The MCC measures how the predictions correlate with the real target values, and their scores range from +1 (always correct) to −1 (always false), and 0 represents a completely random prediction. An MCC score of more than 0.5 was considered to be acceptable as this corresponds to more than 75% accuracy in balanced data. An AUC close to 1 will be a perfect test and an AUC close to 0.5 will be considered as poor tests. Youdin index was calculated to summarize the performance of the prediction tests. Its value ranges from −1 to 1, positive values indicate that there are no false positives or false negatives, i.e. the test is perfect and *vice versa*. Moreover, pair-wise combination analysis was also performed to explore which of the combinations gives best results.

### Structural analysis

#### IL10RA protein modeling and validation

Only short-length three-dimensional (3D) structure of IL10RA protein and its homologous templates were available in PDB database (http://www.rcsb.org/); Therefore, the 3D structure of IL10RA protein has been predicted by iterative threading assembly refinement algorithm (I-TASSER) Standalone Package (Version 1.1). It is an on-line platform that implements algorithms for protein structure and function predictions (Yang et al., 2015). The initial models generated were subjected to energy minimization using the “steepest descent” technique to eliminate bad contacts between protein atoms. Stereo-chemical quality of minimized models was analyzed, using procheck tool (Laskowski et al., 2001).

#### Protein stability analysis

To predict the effects of mutations on the stability of IL10RA protein at three dimensional structure level, DUET (Pires et al., 2014), an integrated computational webserver, was used. The 4-letter code of the crystal structure (1Y6K), chain identifier as well as the residue position of mutation site, residues codes of wild-type and mutant in one-letter format were provided as an input for this server. The collective predictions of SDM (Site Directed Mutator) and mCSM (mutation Cutoff Scanning Matrix) methods are obtained in a non-linear regression fashion. Predictions reveal variation in Gibbs free energy (DDG) wherein positive values denote stabilized mutations and *vice versa*.

#### Mutant models construction

All nine mutated models of IL10RA were built by Swiss Model-Expasy; Wild-type IL10RA model developed with the help of I-Tasser was used as a template for all mutants. A collection of 50 models for each of nine mutated model were generated and subsequently the best model was selected based on the Procheck results.

### Docking

Molecular docking of IL10RA (wildtype or mutant forms) and IL10 molecules was executed using Hex protein docking server. The IL10 ligand molecule's structure was retrieved from the protein database (PDB ID: 2ILK). Maximum rotational increments for receptor and ligand sampling were allowed by setting the angle to 180°. The steric scan (*N* = 20) phase of the docking calculation was performed at 53 intermolecular separations in ± steps at 0.75 Å. The final search (*N* = 25) phase was applied to the highest scoring scan orientations in steps of 0.75/2 Å. Among the 500 clusters, only best 2,000 orientations were retained for viewing out of 10,000 lowest docking energy scores.

### Secondary analysis

#### Conserved regions

The IL10RA protein sequence was searched for homologous sequences using PSI-BLAST. Then the position-specific conservation scores of amino acid variants were determined using ConSurf web server, which calculates the evolutionary conservation of amino acid positions in proteins by means of an ML algorithms empirical or Bayesian inference (Ashkenazy et al., 2010). The conservation scores are ranked by a distinct scale of nine grades for visualization from the most conserved positions colored maroon (grade 9), through intermediately conserved positions colored white (grade 5), to the most variable positions colored turquoise (grade 1).

### Post translational modifications

#### NetPhos: tyrosine phosphorylation site identification

The NetPhos 2.0 server produces neural network predictions for phosphorylation sites in eukaryotic proteins. Neural networks can classify even non-linear and highly complex biological sequence patterns, wherein correlations among positions are important. The network retains the ability to generalize and recognize similar, but non-identical patterns. Artificial neural networks have been widely employed in biological sequence analysis (Blom et al., 2004). The server demands the protein sequence in FASTA format and produces the analyzed report on the cyber platform. Output score ranges from 0.000 to 1.000; scores greater than 0.500 are considered as above the threshold value. A high score indicates increased confidence of the prediction.

### Gene network prioritization (string)

STRING is a database of predicted and known protein interactions, available at http://string-db.org/. The interactions include indirect (functional) and direct (physical) associations which are derived from high-throughput experiments, conserved co-expression, genomic context and previous knowledge. String quantitatively integrates the interaction data from these sources and transfer information between the organisms. The database currently covers 1133 organisms and 5,214,234 proteins. STRING understands a variety of protein names and accessions numbers.

## Results

### Selection of mutations and pathogenicity predictions

A total of 15 mutations causative to early onset IBD (<5 years) were extracted from published research reports and were further used in protein modeling, structural deviation analysis and functional investigations (Table 1). Three clinically significant point mutations, c.537G>A (Yanagi et al., 2016), c.634C>T and c.1291G>T (Lee et al., 2014; Mohammed et al., 2017) were excluded from our structural analysis; as they were reported to cause splicing aberration and pre-mature stop codons, respectively. Table 1 shows that IL10RA mutations are not just specific to one exon. However, out of total 7 exons, exon 3 mutations (6/15; 40%) have been frequently reported compared to other exons like exon 2 (2/15;13.3%), exon 4 (3/15; 20%), exon 5 (1/15; 6.66%), exon 6 (1/15; 6.66%) and exon 7 (2/15;13.3%). Exons 1–5 encodes bulk of the extracellular domain (total 233 amino acids), and exon 6 encodes extracellular domain (230 and 233 aa), transmembrane domain (in between 234 and 256 aa) and cytosolic domain region (257–270 aa) and exon 7 encodes cytosolic domain region (in between 257 and 578 aa) of IL10RA protein. Taken together, SIFT, PolyPhen 2.0, CADD, PROVEAN and Condel methods could predict 79–86% of IL10RA-IBD mutations as deleterious, while the deleterious prediction rate of FATHMM and LRT algorithms is 14 and 28% mutations, respectively. This difference in output suggests that combining scores from different tools may considerably increase the predictive accuracy for determining the functional impact of a given genetic mutation.

**Table 1:** List of IBD causal IL10RA genetic mutations and their pathogenicity prediction scores.

**Nucleotide change**	**Exon**	**Aminoacid change**	**Mutation ID**	**SIFT**	**PolyPhen**	**CADD**	**FATHMM**	**LRT**	**MetaLR**	**MetaSVM**	**PROVEAN**	**Condel**	**References**
c.133T>G	2/7	W45G	–	0	1	4.8	−4.77	1	0.62	1.10	3.33	−11.38	Beser et al., 2015
c.170A>g	2/7	Y57C	rs201643277	0	0.998	4.7	−2.69	1	0.53	0.85	3.275	−7.75	Kotlarz et al., 2012
c.205T>C	3/7	W69R	–	0	1	4.8	−1.03	0.99	0.54	0.55	3.223	−5.16	Shim and Seo, 2014
c.251C>T	3/7	T84I	rs137853580	0	0.998	4.2	−1.18	0.99	0.55	0.32	3.165	−4.66	Glocker et al., 2009; Mao et al., 2012
c.272A>G	3/7	Y91C	–	0	1	3.9	−0.78	0.99	0.14	−0.11	2.64	−4.29	Shim et al., 2013
c.301C>T	3/7	R101W	rs368287711	0	1	6.3	−1.1	0.99	0.62	0.23	3.075	−6.47	Kotlarz et al., 2012; Mao et al., 2012
c.349C>T	3/7	R117C	rs759537444	0	1	7.7	−0.79	1	0.22	0.12	2.51	−4.93	Kotlarz et al., 2012
c.350G>A	3/7	R117H	rs199989396	0	1	7.0	−0.78	1	0.22	0.06	2.51	−2.89	Shim et al., 2013
c.374T>G	4/7	L125R	–	0	0.998	6.0	0.13	0.99	0.02	−0.73	2.51	−4.62	Engelhardt et al., 2013
c.421G>A	4/7	G141R	rs137853579	0.02	1	3.7	0.66	1	0.02	−0.84	2.455	−3.43	Glocker et al., 2009
c.506T>C	4/7	I169T	rs369219156	0.04	0.602	4.4	0.62	1	0.20	−0.83	2.36	−2.84	Kotlarz et al., 2012
c.670A>G	5/7	I224V	rs2228055	0.49	0.002	−2.1	1.04	0.99	0.08	−0.98	0.69	−0.17	Moran et al., 2013
c.784C>T	6/7	R262C	rs149491038	0	0.88	6.8	0.29	0.99	0.11	−0.85	2.33	−4.59	Begue et al., 2011; Shim et al., 2013
c.1234C>T	7/7	R412W	rs143538561	0.01	0.326	4.3	1.96	0.99	0.02	−1.00	0.69	−2.11	Kelsen et al., 2015
c.1235G>A	7/7	R412Q	rs117423374	1	0	0.5	1.99	0.99	0.02	−1.02	−1.35	1.27	Kelsen et al., 2015

*Two clinically significant point mutations, c.537G>A (Yanagi et al., 2016) and c.1291G>T (Lee et al., 2014; Mohammed et al., 2017) were excluded, as they were reported to cause splicing aberration and pre-mature stop codon, respectively*.

### Concordance analysis report

The concordance analysis reported; p.W45G (c.133T>G) and p.Y57C (c.170A>G) were rank-I mutations revealing them to be highly deleterious. Six mutations (p.W69G, p.T84I, p.Y91C, p.R101W, p.R117C, and p.R117H) were categorized as rank-II (Deleterious). Seven mutations (p.L125R, p.G141R, p.R262C, p.I169T, p.I224V, p.R412W, and p.R412Q) were of least significance, hence graded as rank-III (Neutral). Highly significant variants in rank-I and II were shortlisted for structural analysis considering their strong association with IBD and consensus scoring scheme. The statistical analysis has revealed significant relation among different tools (Table 2). CADD outperformed Provean, PolyPhen, and Condel in most of the statistical measures namely Youden's index, MCC, AUC and specificity. Pair-wise combinations results showed improved performance with PolyPhen + CADD exhibiting optimal balance between sensitivity and specificity, due to its high accuracy (S1 Table).

**Table 2:** Specificity, sensitivity analysis report of IL10 RA mutation predictions by different computational methods.

	**Sensitivity**	**Specificity**	**AUC**	**Accuracy**	**MCC**	**Y-Index**
SIFT	71.43	76.92	0.695	59.26	0.19	0.4835
Polyphen	66.29	100	0.799	70.37	0.41	0.6429
CADD	85.71	100	0.923	92.59	0.86	0.8571
FATHMM	50	100	0.599	55.56	0.27	0.5
LRT	100	38.46	0.5	48.15	−0.03	0.3846
SVM	42.86	100	0.621	70.37	0.52	0.4286
META LR	35.71	92.31	0.599	59.26	0.19	0.2802
Provean	64.29	92.31	0.753	74.07	0.48	0.5659
Condel	57.14	100	0.799	59.26	0.2	0.5714

*Dark color indicates higher score and vice versa*.

### Identification of functional mutations in conserved motifs

**Figure 1:**
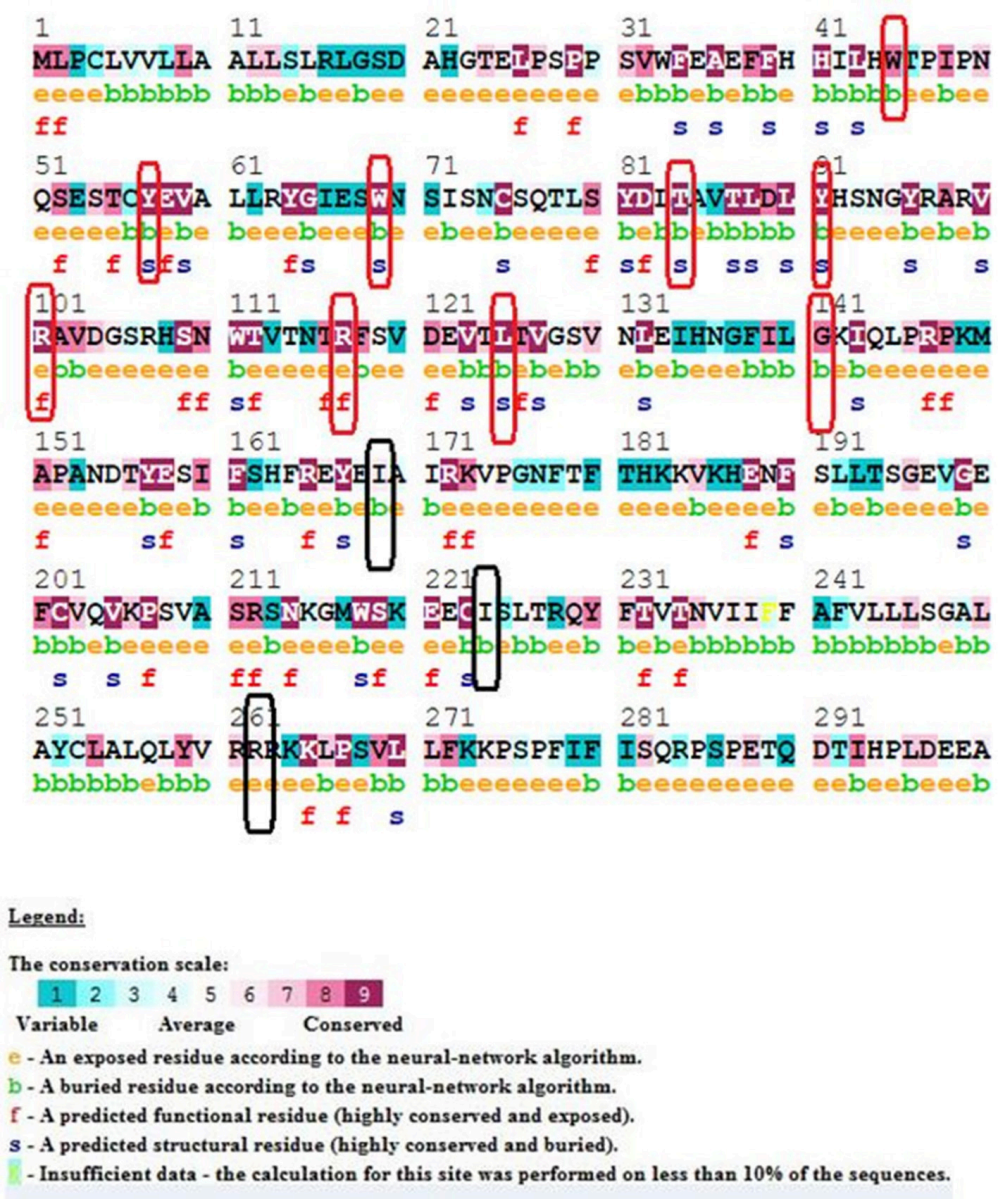
ConSurf Output of selected IL10RA mutants. Wild-type sequence of IL10RA with its conservation scale; highly conserved residues in red rectangle; average conserved residues in black rectangles.

Our ConSurf results have shown that a total of 10 (out of 15) mutations corresponding to W45G, Y57C, W69R, T84I, Y91C, R101W, R117C, R117H, L125R, and G141R are located in conserved regions in IL10RA protein sequence. Of which, mutations corresponding to Arg101Trp and Arg117Cyt/His are highly conserved and surface exposed residues and may likely to have functional influence on IL10RA protein. The remaining eight mutants (from the above 10 conserved residues) Y57C, T84I, Y91C, and G141R are found to be highly conserved but in buried state in both wild type and mutant forms of the protein. The p.I169T, p.I224V, p.R262C and R412W/Q are found to be averagely conserved residues (Figure 1).

### Modeling and validation

**Figure 2:**
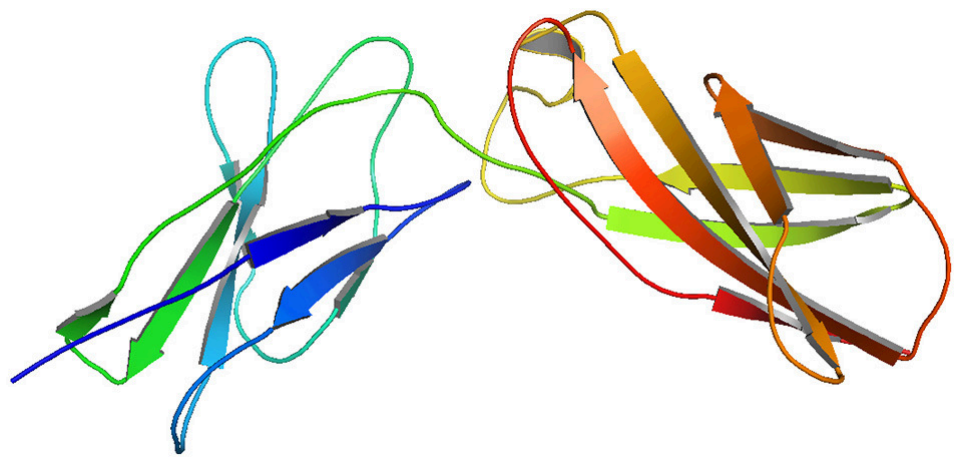
I-Tasser generated molecular model of IL10RA.

Though, the PDB sourced IL10RA (1Y6K; 22–235 residues) had sequence homology to build a 3D model, it was of partial length. For this reason we constructed 3D models of IL10RA by using I-Tasser web server (Figure 2). A total of five structures were predicted for IL10RA. Only best structures were selected based on their maximum C-score (−3.64), TM-scores (0.32 ± 0.10) which reflects that the correct topology of the model built. The selected model was further used as template to build mutant protein models using Swiss Model-Expasy. We have built three dimensional models for 12 out of 15 mutations spanning the extracellular domain region of IL10RA protein. All these protein models were saved in PDB format and visualized using by Pymol program.

#### Quality of the 3D models

After the energy minimization of 3D models, stereochemical quality of the structures was validated by PROCHECK server. The results of the PROCHECK analysis indicated that a relatively low percentage of residues have phi/psi angles in the disallowed regions suggesting the acceptability of Ramachandran plots for proteins. The percentage of residues in the allowed/core region were found to be 98.9, 99.2, 98.8, 97.2, 92.5, 96.2, 98.3 and disallowed region were found to be 1.1, 0.8, 1.2, 2.8, 2.8, 7.5, and 3.8 for wild-type and mutated IL10RA respectively. The ERRAT score for wild-type and all mutant models is >50, this confirms the high quality of the model.

#### Structural deviation analysis

**Figure 3:**
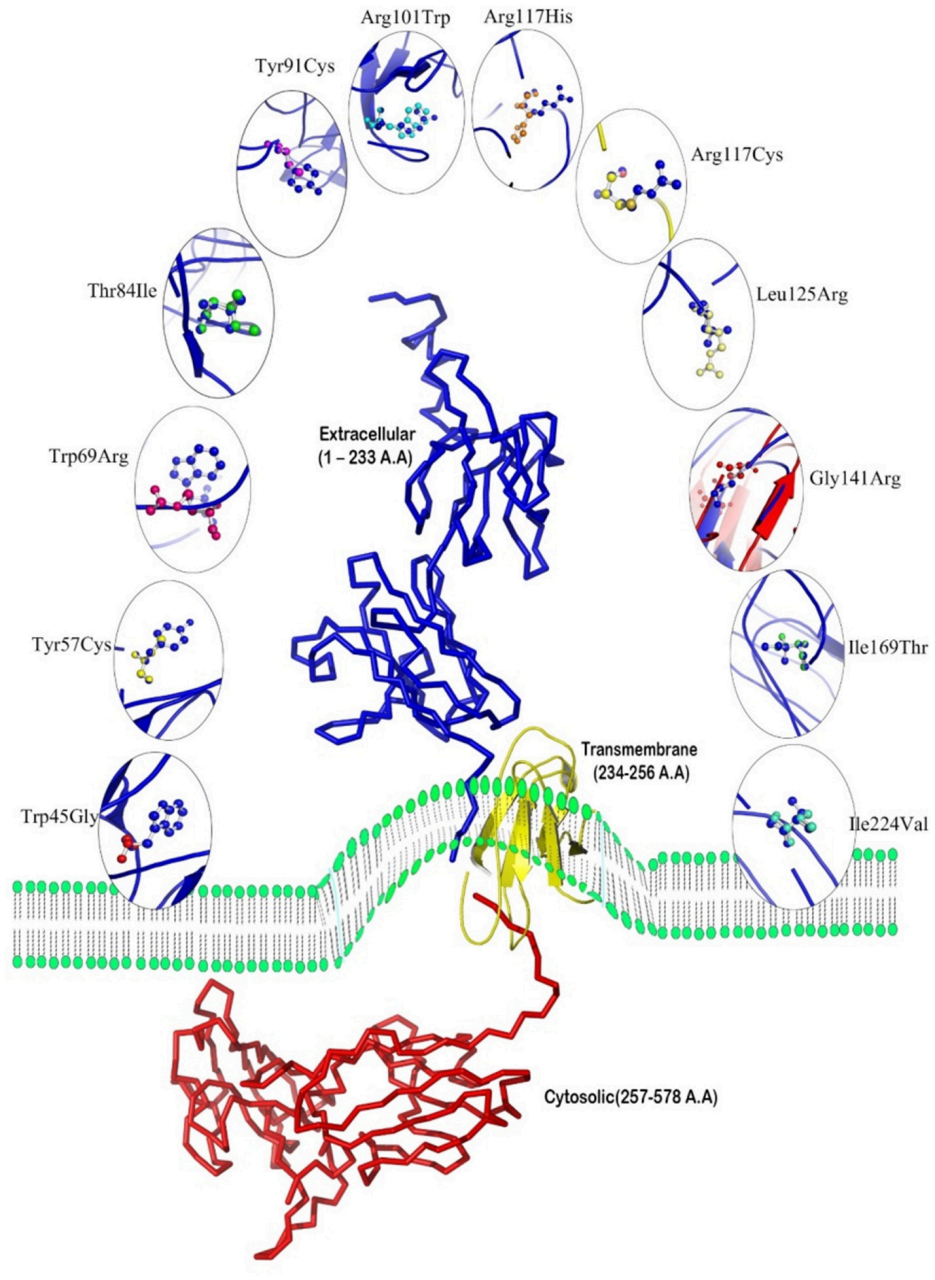
Overview of 12 mutated models with superpose center figure. The IL10RA models represented in cartoon and mutated amino acids are represented non-green colored in sticks.

Superimposition of 12 mutant models and wild-type IL10RA indicates both structural similarities and differences among them. The close homology between template and targets is revealed by RMSD score of less than 0.2 Å (Figure 3). The Structural Distance Measure (SDM) score (Cut off 3.5 Å) for all models fall within the range of zero, which indicates that the sequence-derived distance of the built models is very small. The Q-score (Cut off 3Å) for all mutant IL10RA models is >1, revealing the fair superimposition of mutant models over template structure.

### Mutation effect on structural stability of IL10RA

The protein stability of 12 mutations in terms of ΔΔG (Gibbs free energy change) using DUET web server was tested. Table 3 reveals that W45G, Y57C, T84I, Y91C, R101W, R117C, R117H, L125R, G141R, and R262 C mutant models have shown the ΔΔG values ranging from −0.22 to −4.017 kcal/Mol. The negative ΔΔG values suggest that the given amino acid substitutions are deleterious to the stability of IL10RA protein (Table 3).

**Table 3:** Structural stability prediction scores, secondary structural features and molecular docking analysis of IL10RA models.

**Protein**	**Mutated A.A**	mCSM^*^ΔΔG (Kcal/mol)	SDM^*^ΔΔG (Kcal/mol)	DUET^*^ΔΔG (Kcal/mol)	**Secondary structure**	**RMSD**	**Binding Energy(BE) with IL10 (KJ/Mol)**	
							**Actual BE**	**Difference BE**
IL10RA	WildType	–	–	–	–	–	−435.89	–
	W45G	−4.308	−5.73	−4.017	Extended beta-strand	0.52	−469.27	−33.38
	T57C	−1.043	1.02	−1.073	H-bonded turn	0.25	−474.84	−38.95
	W69R	−1.193	−1.86	−1.212	H-bonded turn	0.24	−409.84	−26.05
	T84I	−0.466	2.1	−0.264	Extended beta-strand	0.22	−456.65	−20.76
	Y91C	−0.284	1.45	−0.099	Loop or irregular	0.28	−477.77	−41.88
	R101W	−0.997	0.55	−1.233	Extended beta-strand	0.01	−386.86	49.03
	R117C	−1.906	2.22	−1.787	Bend	0.09	−437.10	−1.21
	R117H	−2.16	1.03	−2.256	Bend	0.15	−394.61	41.28
	L125R	−0.452	0.74	−0.22	Bend	0.25	−469.27	−33.38
	G141R	−1.016	1.28	−0.924	Isolated beta-bridge	0.48	−460.71	−24.82

* *RMSD > 0.5 alters protein ionic interaction; $ΔΔG > 0 destabilizes the protein structure*.

### Docking

**Figure 4:**
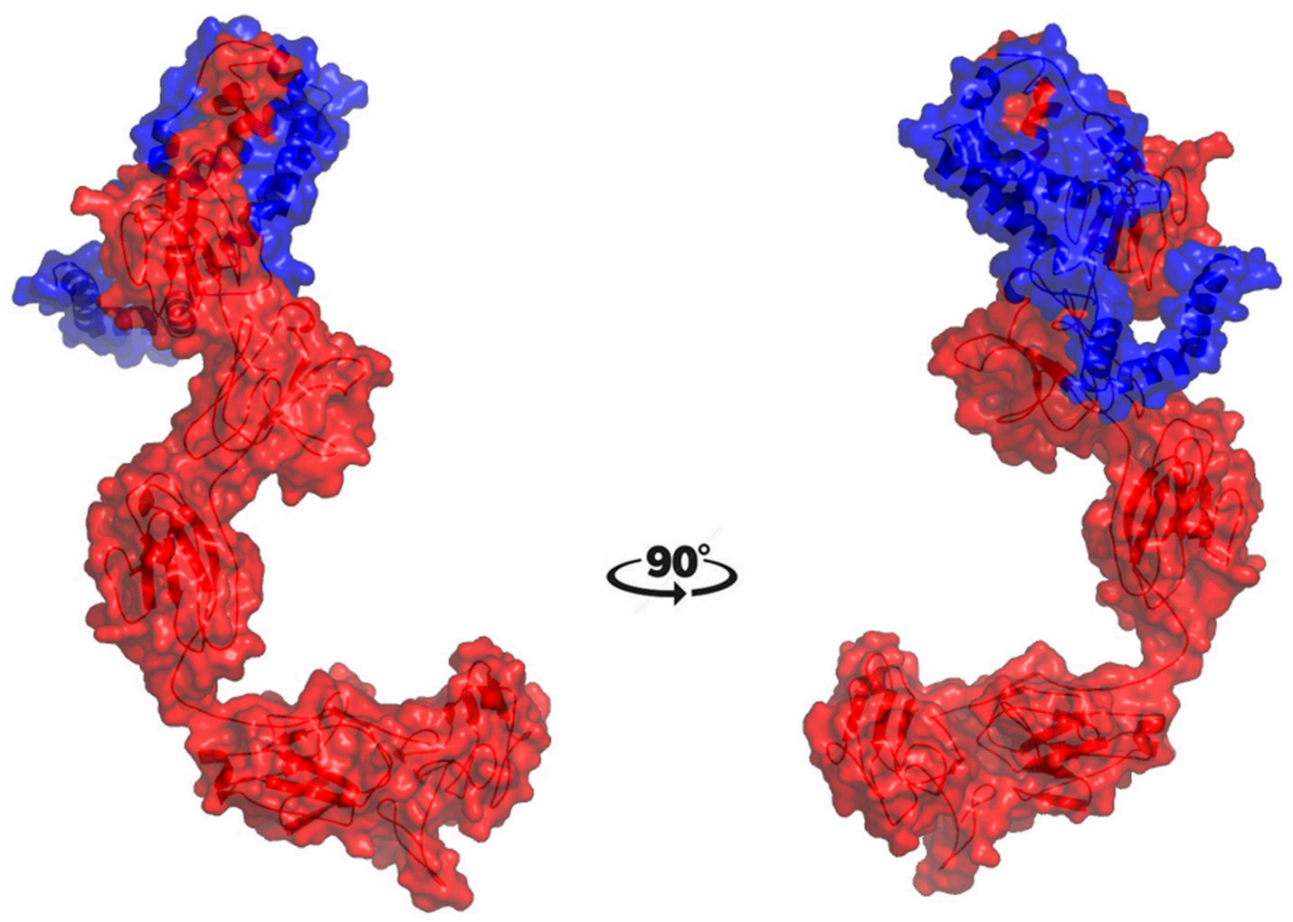
Stereo surface docking view of IL10RA wildtype (RED color) with IL10 (Blue).

**Figure 5:**
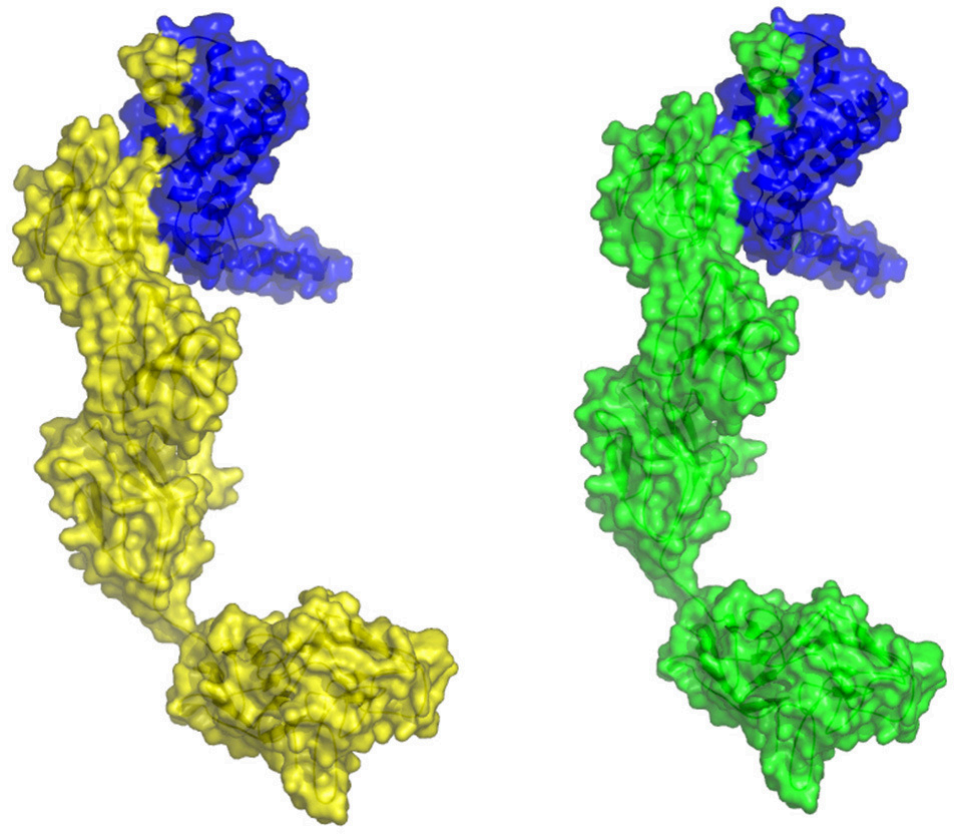
Molecular surface docking view of selected mutant models. Mutant IL10RA-Y91C (Yellow in color) with IL10 (Blue in color); and mutant IL10RA-R101W (Green in color) with IL10 (Blue in color); Based on Highest to lowest difference in binding energies compared with wildtype docking complex.

The molecular docking procedure was performed with mutations which are mapped to extra cellular regions. The last two columns of Table 3 revealed significant changes in binding energies between mutant IL10RA and IL10 proteins. The interaction energy of wild-type IL10RA and IL10 was found to be −435.89 kcal/mol. However, the degree of affinity between IL10RA mutants and IL10 were varying; Striking differences in binding energies were witnessed for R101W (49.03 kcal/mol), Y91C (−41.88 kcal/mol), R117H (41.28 kcal/mol) and Y57C (−38.95 kcal/mol) amino acid substitution forms. This difference in interaction energy is likely to alter the efficacy of IL10 binding to IL10RA (Figures 4, 5).

### Protein-protein molecular interaction studies

**Figure 6:**
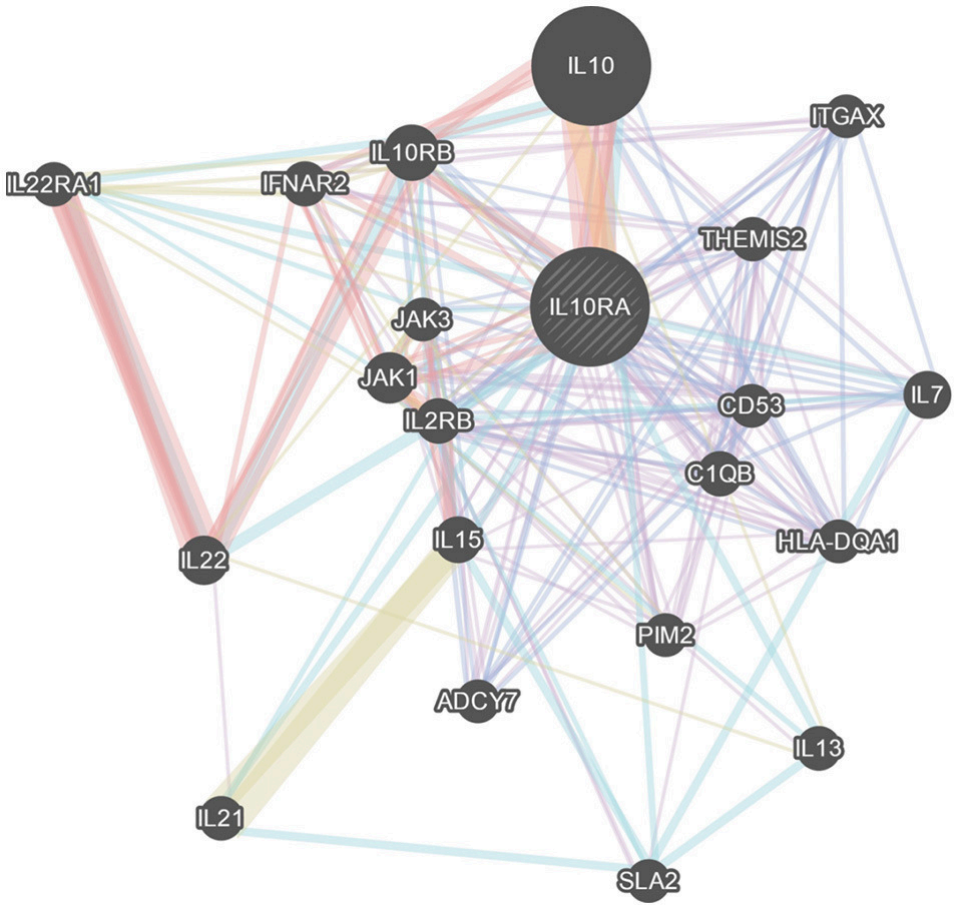
GeneMania network analysis of IL10RA. Showing its strong network of interactions with important genes in immune system regulation.

The STRING server result has revealed the direct interaction of IL10RA protein with IL10, JAK1, and IL10RB in terms of high confidence score (>0.990) based on text-mining, database and experiments. All the three proteins retain strong network edges of their predicted functional associations with IL10RA and were displayed to be involved in mediating signal transduction. From the predicted interaction network it is evident that IL10 is one of the strongest interacting partners (c score¼ > 0.999), which, in conjunction with JAK1, leads to nuclear translocation of STAT3 and gene transcription (Figure 6).

### Post-translational modification report

**Figure 7:**
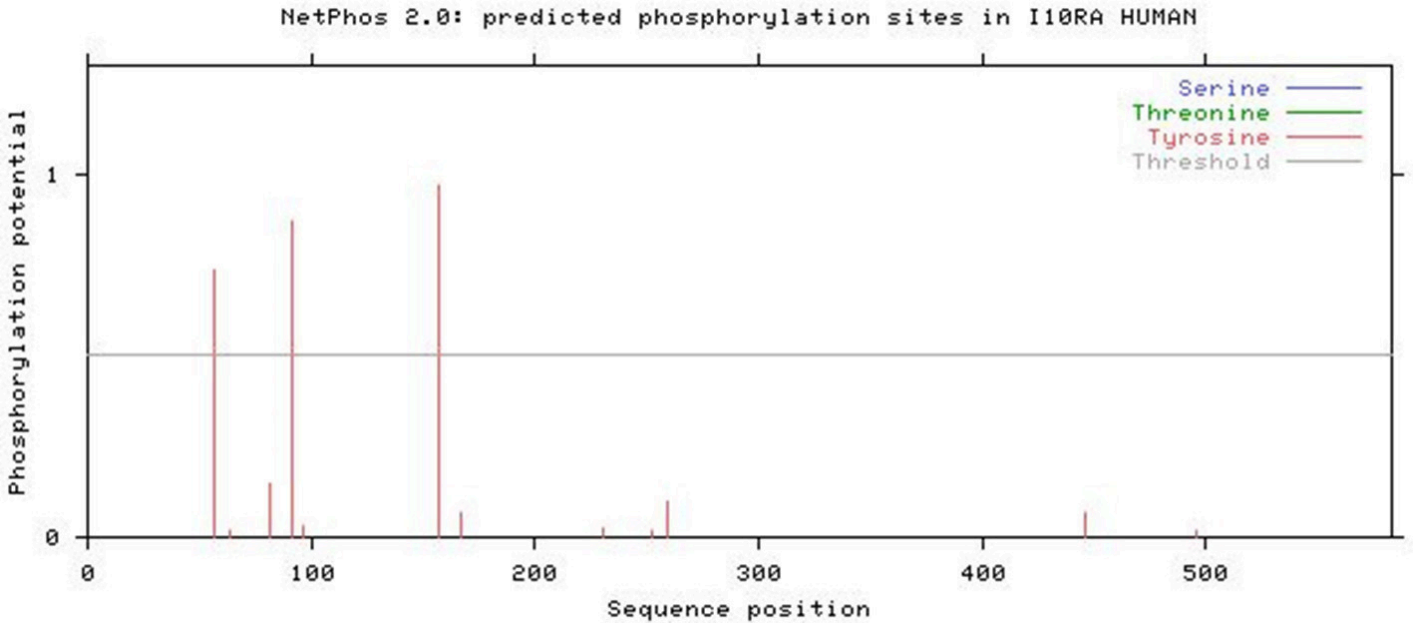
NetPhos 2.0 result showing post-translational modification.

The NetPhos 2.0 results revealed that IL10RA protein has three phosphorylation sites (threshold score of >0.500) corresponding to Tyrosine residue at 57, 91, and 157 positions in the protein sequence. Figure 7 illustrates the threshold value and predicted phosphorylation sites of the protein along with their prediction score.

## Discussion

The human IL10RA gene is located on chromosome 11q23.3 region with 7 exons. A 3,695 bp length m-RNA encodes the functional IL10RA protein with 578 aa (63.0 kDa, pI 5.38). Nonetheless, alternative splicing of its mRNA yields polynucleotides chains of smaller lengths i.e., 2,275 and 1,179 bps with 558 and 429 amino acids, respectively. Hitherto approximately 600 variants located in coding, non-coding and regulatory regions of human IL10RA gene are described in Ensembl database. With the advent of high throughput sequencing practices, the number of genetic mutations is growing with time in an exponential pattern. Therefore, delineating those mutations which imposes specific functional and structural alterations is an important yet un-met area of research concerning IL10RA. The present study gains significance by predicting the potentially deleterious IL10RA mutations and their corresponding phenotypes. This aids in narrowing down the number of mutants analyzed in association with diseases that are most likely to alter gene function.

As a matter of fact, among all the known IL10RA mutants' causative to different health disorders, non-synonymous mutations, which are located in protein-coding region have the potential to alter the protein molecule. However, genetic mutations in non-coding regions may also have an impact on gene splicing, non-coding RNA or transcription factor binding. The interaction site of IL10RA is comprised of 23 residues displaying five receptors segments designated as L2-L6; Residues 62–69 (L2), 94–97 (L3), 113–122 (L4), 163–164 (L5), and 208–214 (L6) (Josephson et al., 2001) which makes contact with three discontinuous peptide segments of IL10 corresponding to helix A, loop AB, and helix F (Josephson et al. referred 62nd residue as 41st and so on, this difference was basically due to incomplete protein transcript crystallized by them).

To identify the deleterious potential of IL10RA mutations we applied diverse approaches, i.e., empirical rule-based algorithm screening and support vector based protein stability predictions. Multiple tools namely SIFT, PolyPhen 2.0, CADD, FATHMM, LRT, MetaLR, MetaSVM, PROVEAN and Condel, are employed to assess the reliability of prediction scores. The computational studies conducted by Doss and Rajith (2012), Shaik et al. (2014), and Banaganapalli et al. (2016) have successfully applied an analogous computational prediction programs for prioritizing the variants in ATM, IDE, and MED12 genes, respectively. The concordant prediction of all the three tools have identified the deleterious potential of 10 missense mutations of IL10RA (p.W45G, p.Y57C, p.W69R, p.T84I, p.Y91C, p.R101W, p.R117C, and p.R117H). The performance of the scores was measured using receiver operating characteristic (ROC) curve and area under the curve (AUC) (S1 Figure). An AUC close to 1 will be a perfect test and an AUC close to 0.5 will be considered as poor tests. We found that, the four prediction tools, CADD, Provean, PolyPhen, and Condel, achieved excellent prediction accuracy (AUC > 0.7). LRT was observed to be with lowest accuracy (AUC = 0.5). Pair-wise combinations revealed that CADD being highly accurate tool, uplifts the performance of other averagely performing tools resulting in five best pairs i.e., CADD + Provean, CADD + PolyPhen, CADD + FATHMM, CADD + SVM, and CADD + Condel.

The functional impact of genetic mutations can be predicted based on the changes it brings in the structural conformation of protein molecule it encodes. IL10 receptors are trans-membrane proteins, therefore, it is difficult to obtain a complete tertiary structural conformation of its mutant and native models through the conventional NMR spectroscopy or X-ray crystallography methods. For this reason, in the present study both mutant and native protein 3D models of IL10 receptors were built by homology modeling and integrative *ab-initio* methods for analyzing secondary structure & the change of protein stability affected by a variation.

Our analysis revealed significant structural deviations between the wild-type and all 12 mutated models of IL10RA, at both whole protein and amino-acid residue levels. Specifically p.R117C and p.R117H that are located on the interaction site “L4” within the D1 region of the extracellular domain of IL10RA. Prior to L4 (residues 113-122) IL10RA protein consists of a “WSXWS-like” sequence motif represented as “HSNWT” amino acids at positions 108-112 dividing β strand G into two equivalent segments (G1 and G2). The side chain oxygen of Ser-109 hydrogen bonds to the amide nitrogen on β strand F (Ala-102). This hydrogen bonding pattern results in two modified wide β bulges (B1 and B2) that contain one (Asn-110) and three (Asn-115, Thr-116, Arg-117) bulged residues, respectively. B2 positions the Cα-Cβ bond of Arg-117 approximately parallel, rather than perpendicular, to β strand G2 so that it can participate in extensive interactions with IL10 in the site I interface. Thus, Arg-117 forms an extensive hydrogen bond/salt bridge with Asp-162, Gln-56, and the main chain carbonyl oxygen of Ser-159 of IL10. Any mutation at this point distorts the expected complementary nature of the ligand and receptor. In mutant p.T84I hydrogen bond of wild type IL10RA between Thr84 and His41 was missing similarly p.R101W disrupted two putative hydrogen bonds between Glu58 and Arg101. These structural changes were reported to abrogate IL10RA phosphorylation induced by IL10, consequently impairs STAT3 activation and suppresses the inflammatory responses (Mao et al., 2012).

The RMSD value for the identical proteins will always be “zero” and its increase reflects the structural deviation between two protein structures which may be due to the disruption of hydrogen bonds, hydrophilic or hydrophobic properties and electro static charges. Altered spatial arrangements of the active sites often negatively influence the binding efficacy with its ligand resulting in immobilized pathway. Owing to the lack of negative-feedback signaling mediated by IL10 perturbs homeostasis of the intestinal immune system (Glocker et al., 2009).

ConSurf analysis has identified that genetic mutations encoding p.Y57C, p.T84I, p.Y91C, p.R101W, p.R117C, p.R117H, and p.G141R are potentially deleterious, as they are located in evolutionarily highly conserved regions of IL10RA protein sequence. The surface accessibility of IL10RA mutants showed a huge drift in the Z-score for p.T84I, p.Y91C, and p.R101W models indicating that they can induce conformational changes in the IL10RA structures. Docking studies explored that the two amino acid residues found in the extracellular domain of IL10RA *viz.*, Thr84 and Arg101 play vital role in the formation of different type of ionic interaction inIL10RA protein for maintaining the stability of its structure. Hydrogen bond existing in between Thr84 and His41 residues in the native IL10RA was disrupted by the p.T84I substitution. The p.R101W variant not only abolished two putative hydrogen bonds between Glu58 and Arg101 present in the native IL10RA, but also caused steric-clashes between Val103 and Trp111, Trp101, and Glu58 (Mao et al., 2012). These structural conformations might interfere with either binding affinity, stability of IL10 or signal transduction.

In conclusion, using comprehensive *in-silico* investigations, our current study confirmed that p.W45G, p.Y57C, p.W69R, p.T84I, p.Y91C, p.R101W, p.R117C, p.R117H, p.L125R, and p.G141R mutations are deleterious to the structure and function of IL10RA protein. Molecular modeling analysis has revealed that p.T84I, p.Y91C, and p.R101W variants may induce changes in the stability of IL10RA protein by altering the solvent accessibility. Altered IL10RA function due to genetic variation and protein expression may also play a critical role in determining the individuals' susceptibility to IBD. Our findings may help to narrow down the number of IL10RA variants to be screened for genetic association studies and to have a better insight about the structural biology of mutated IL10RA proteins deciphering IL10RA dysfunction.

## Author contributions

FA-A: Designing the protein analysis work methodology, results explanation, and funding acquisition. KM: Extracted genetic data, performed experiments, analysis of results, and manuscript preparation. SS: Performed experiments, generated and analyzed data. BB: Software testing, data extraction, figures, and tables generation. KN: Analyzed the results and critically revised the manuscript. NS: Study design, results analysis, explanation, and manuscript preparation.

### Conflict of interest statement

The authors declare that the research was conducted in the absence of any commercial or financial relationships that could be construed as a potential conflict of interest.
