# Supplementary material for: Computational Protein Phenotype Characterization of IL10RA Mutations Causative to Early Onset Inflammatory Bowel Disease (IBD)
Source: Front Genet. 2018 Apr 27;9:146. doi: 10.3389/fgene.2018.00146 (PMC5934427; doi:10.3389/fgene.2018.00146)
Supplement: Supplementary file 1 [file Table_1.DOCX]

Supplementary Material

**Computational Protein Phenotype Characterization of IL10RA Mutations Causative to Early Onset Inflammatory Bowel Disease (IBD)**

Fahad Ahmed Al-Abbasi ^¶^, Kaleemuddin Mohammed ^¶^*, Saida Sadath, Babajan Banaganapalli, Khalidah Nasser, Noor Ahmad Shaik*

*** Correspondence:** Dr. Noor Ahmad Shaik [noorahmadh@gmail.com](mailto:noorahmadh@gmail.com) Kaleemuddin Mohammed [kaleem_kamran111@yahoo.com](mailto:kaleem_kamran111@yahoo.com)

^¶^ These authors (FA and KM) contributed equally to this work and prefers to be considered as first authors.

1. **Details of the formulas used in the analysis:**

**True positive (TP)** = the number of **IBD-Causal IL10RA GVs predicted as ‘deleterious’** by the *in-silico* tool.

**False positive (FP)** = the number of **IL10RA common polymorphism predicted as ‘deleterious’** by the *in-silico* tool.

**True negative (TN)** = the number **IL10RA common polymorphism predicted as ‘benign’** by the *in-silico* tool.

**False negative (FN)** = the number of **IBD-Causal IL10RA GVs predicted as ‘benign’** by the *in-silico* tool.

Formulas used:

$$\mathrm{Sensitivity}=\frac{\mathrm{TP}}{(TP+FN)}$$

$$\mathrm{Specificity}=\frac{\mathrm{TN}}{(TN+FP)}$$

Youden's index = sensitivity + specificity – 1

$$Accuracy=\frac{(TP+TN)}{(TP+TN+FP+FN)}$$

$$\mathrm{MCC}=\frac{(TP x TN) - (FP x FN)}{\sqrt{((TP + FP) (TP + FN) (TN + FP) (TN + FN))}}$$

1. Supplementary Figure:

S1 Fig. ROC (receiver operating characteristic) curves illustrating the performance of various prediction algorithms used in the *in-silico* analysis of IL10RA genetic variants


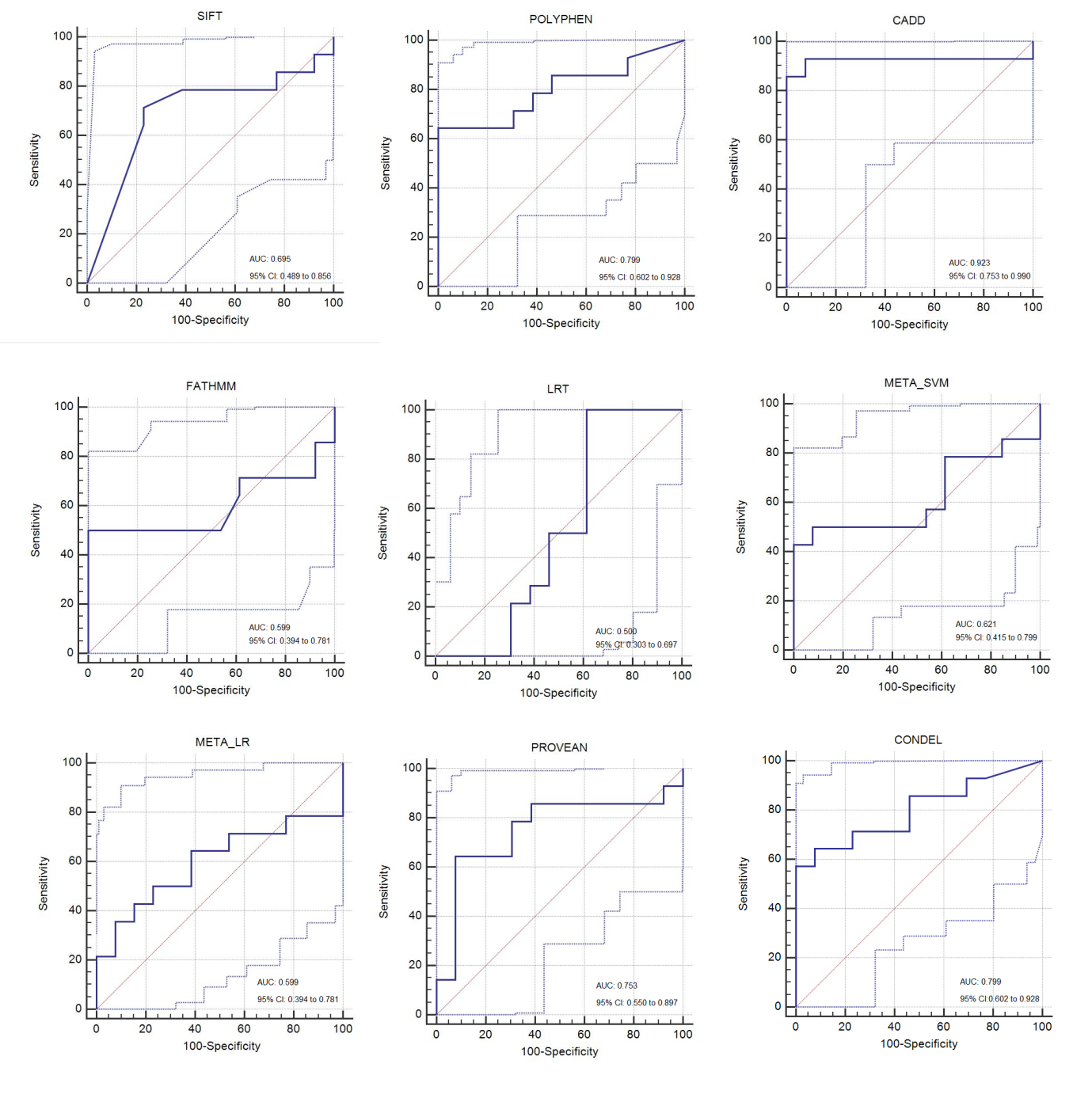


1. Supplementary Tables:

S1 Table. Pair-wise combination of different *in-silico* tools measured by various statistical values like Youden’s Index, Accuracy and Mathews coefficient correlation (MCC) etc.

| AUC | | | | | | | | | |
| --- | --- | --- | --- | --- | --- | --- | --- | --- | --- |
|  | SIFT | Polyphen | CADD | FATHMM | LRT | SVM | META LR | Provean | Condel |
| SIFT |  | 74.7 | 80.9 | 64.7 | 59.75 | 65.8 | 64.7 | 72.4 | 74.7 |
| Polyphen |  |  | 86.1 | 69.9 | 64.95 | 71 | 69.9 | 77.6 | 79.9 |
| CADD |  |  |  | 76.1 | 71.15 | 77.2 | 76.1 | 83.8 | 86.1 |
| FATHMM |  |  |  |  | 54.95 | 61 | 59.9 | 67.6 | 69.9 |
| LRT |  |  |  |  |  | 56.05 | 54.95 | 62.65 | 64.95 |
| SVM |  |  |  |  |  |  | 61 | 68.7 | 71 |
| META LR |  |  |  |  |  |  |  | 67.6 | 69.9 |
| Provean |  |  |  |  |  |  |  |  | 77.6 |
| Condel |  |  |  |  |  |  |  |  |  |

| SENSITIVITY | | | | | | | | | | | | | | | | | | | | | | | | | | | | | | | | | | | | | |  |  |
| --- | --- | --- | --- | --- | --- | --- | --- | --- | --- | --- | --- | --- | --- | --- | --- | --- | --- | --- | --- | --- | --- | --- | --- | --- | --- | --- | --- | --- | --- | --- | --- | --- | --- | --- | --- | --- | --- | --- | --- |
|  | | SIFT | | | | Polyphen | | | | CADD | | | | FATHMM | | | | LRT | | | | SVM | | | | | | META LR | | | | Provean | | | Condel | | |  |  |
| SIFT | |  | | | | 68.86 | | | | 78.57 | | | | 60.715 | | | | 85.715 | | | | 57.145 | | | | | | 53.57 | | | | 67.86 | | | 64.285 | | |  |  |
| Polyphen | |  | | | |  | | | | 76 | | | | 58.145 | | | | 83.145 | | | | 54.575 | | | | | | 51 | | | | 65.29 | | | 61.715 | | |  |  |
| CADD | |  | | | |  | | | |  | | | | 67.855 | | | | 92.855 | | | | 64.285 | | | | | | 60.71 | | | | 75 | | | 71.425 | | |  |  |
| FATHMM | |  | | | |  | | | |  | | | |  | | | | 75 | | | | 46.43 | | | | | | 42.855 | | | | 57.145 | | | 53.57 | | |  |  |
| LRT | |  | | | |  | | | |  | | | |  | | | |  | | | | 71.43 | | | | | | 67.855 | | | | 82.145 | | | 78.57 | | |  |  |
| SVM | |  | | | |  | | | |  | | | |  | | | |  | | | |  | | | | | | 39.285 | | | | 53.575 | | | 50 | | |  |  |
| META LR | |  | | | |  | | | |  | | | |  | | | |  | | | |  | | | | | |  | | | | 50 | | | 46.425 | | |  |  |
| Provean | |  | | | |  | | | |  | | | |  | | | |  | | | |  | | | | | |  | | | |  | | | 60.715 | | |  |  |
| Condel | |  | | | |  | | | |  | | | |  | | | |  | | | |  | | | | | |  | | | |  | | |  | | |  |  |
| SPECIFICITY | | | | | | | | | | | | | | | | | | | | | | | | | | | | | | | | | | | | |  |  |  |
|  | | | SIFT | | | | Polyphen | | | | CADD | | | | FATHMM | | | | LRT | | | | SVM | | | META LR | | | | Provean | | | | Condel | | |  |  |  |
| SIFT | | |  | | | | 88.46 | | | | 88.46 | | | | 88.46 | | | | 57.69 | | | | 88.46 | | | 84.615 | | | | 84.615 | | | | 88.46 | | |  |  |  |
| Polyphen | | |  | | | |  | | | | 100 | | | | 100 | | | | 69.23 | | | | 100 | | | 96.155 | | | | 96.155 | | | | 100 | | |  |  |  |
| CADD | | |  | | | |  | | | |  | | | | 100 | | | | 69.23 | | | | 100 | | | 96.155 | | | | 96.155 | | | | 100 | | |  |  |  |
| FATHMM | | |  | | | |  | | | |  | | | |  | | | | 69.23 | | | | 100 | | | 96.155 | | | | 96.155 | | | | 100 | | |  |  |  |
| LRT | | |  | | | |  | | | |  | | | |  | | | |  | | | | 69.23 | | | 65.385 | | | | 65.385 | | | | 69.23 | | |  |  |  |
| SVM | | |  | | | |  | | | |  | | | |  | | | |  | | | |  | | | 96.155 | | | | 96.155 | | | | 100 | | |  |  |  |
| META LR | | |  | | | |  | | | |  | | | |  | | | |  | | | |  | | |  | | | | 92.31 | | | | 96.155 | | |  |  |  |
| Provean | | |  | | | |  | | | |  | | | |  | | | |  | | | |  | | |  | | | |  | | | | 96.155 | | |  |  |  |
| Condel | | |  | | | |  | | | |  | | | |  | | | |  | | | |  | | |  | | | |  | | | |  | | |  |  |  |
| ACCURACY | | | | | | | | | | | | | | | | | | | | | | | | | | | | | | | | | | | | | | | |
|  | | | | | SIFT | | | | Polyphen | | | | CADD | | | | FATHMM | | | | LRT | | | | SVM | | | | META LR | | | | Provean | | | Condel | | | |
| SIFT | | | | |  | | | | 64.82 | | | | 75.93 | | | | 57.41 | | | | 53.71 | | | | 64.82 | | | | 59.26 | | | | 66.67 | | | 59.26 | | | |
| Polyphen | | | | |  | | | |  | | | | 81.48 | | | | 62.97 | | | | 59.26 | | | | 70.37 | | | | 64.82 | | | | 72.22 | | | 64.82 | | | |
| CADD | | | | |  | | | |  | | | |  | | | | 74.08 | | | | 70.37 | | | | 81.48 | | | | 75.93 | | | | 83.33 | | | 75.93 | | | |
| FATHMM | | | | |  | | | |  | | | |  | | | |  | | | | 51.86 | | | | 62.97 | | | | 57.41 | | | | 64.82 | | | 57.41 | | | |
| LRT | | | | |  | | | |  | | | |  | | | |  | | | |  | | | | 59.26 | | | | 53.71 | | | | 61.11 | | | 53.71 | | | |
| SVM | | | | |  | | | |  | | | |  | | | |  | | | |  | | | |  | | | | 64.82 | | | | 72.22 | | | 64.82 | | | |
| META LR | | | | |  | | | |  | | | |  | | | |  | | | |  | | | |  | | | |  | | | | 66.67 | | | 59.26 | | | |
| Provean | | | | |  | | | |  | | | |  | | | |  | | | |  | | | |  | | | |  | | | |  | | | 66.67 | | | |
| Condel | | | | |  | | | |  | | | |  | | | |  | | | |  | | | |  | | | |  | | | |  | | |  | | | |
| Youden’s Index | | | | | | | | | | | | | | | | | | | | | | | | | | | | | | | | | | | | | |  |  |
|  | | | SIFT | | | | Polyphen | | | | CADD | | | | FATHMM | | | | LRT | | | | SVM | | | META LR | | | | Provean | | | | Condel | | | |  |  |
| SIFT | | |  | | | | 0.3 | | | | 0.525 | | | | 0.23 | | | | 0.08 | | | | 0.355 | | | 0.19 | | | | 0.335 | | | | 0.195 | | | |  |  |
| Polyphen | | |  | | | |  | | | | 0.635 | | | | 0.34 | | | | 0.19 | | | | 0.465 | | | 0.3 | | | | 0.445 | | | | 0.305 | | | |  |  |
| CADD | | |  | | | |  | | | |  | | | | 0.565 | | | | 0.415 | | | | 0.69 | | | 0.525 | | | | 0.67 | | | | 0.53 | | | |  |  |
| FATHMM | | |  | | | |  | | | |  | | | |  | | | | 0.12 | | | | 0.395 | | | 0.23 | | | | 0.375 | | | | 0.235 | | | |  |  |
| LRT | | |  | | | |  | | | |  | | | |  | | | |  | | | | 0.245 | | | 0.08 | | | | 0.225 | | | | 0.085 | | | |  |  |
| SVM | | |  | | | |  | | | |  | | | |  | | | |  | | | |  | | | 0.355 | | | | 0.5 | | | | 0.36 | | | |  |  |
| META LR | | |  | | | |  | | | |  | | | |  | | | |  | | | |  | | |  | | | | 0.335 | | | | 0.195 | | | |  |  |
| Provean | | |  | | | |  | | | |  | | | |  | | | |  | | | |  | | |  | | | |  | | | | 0.34 | | | |  |  |
| Condel | | |  | | | |  | | | |  | | | |  | | | |  | | | |  | | |  | | | |  | | | |  | | | |  |  |

| MCC | | | | | | | | | |
| --- | --- | --- | --- | --- | --- | --- | --- | --- | --- |
|  | SIFT | Polyphen | CADD | FATHMM | LRT | SVM | META LR | Provean | Condel |
| SIFT |  | 56.32 | 67.03 | 49.175 | 43.405 | 45.605 | 38.185 | 52.47 | 52.745 |
| Polyphen |  |  | 75 | 57.145 | 51.375 | 53.575 | 46.155 | 60.44 | 60.715 |
| CADD |  |  |  | 67.855 | 62.085 | 64.285 | 56.865 | 71.15 | 71.425 |
| FATHMM |  |  |  |  | 44.23 | 46.43 | 39.01 | 53.295 | 53.57 |
| LRT |  |  |  |  |  | 40.66 | 33.24 | 47.525 | 47.8 |
| SVM |  |  |  |  |  |  | 35.44 | 49.725 | 50 |
| META LR |  |  |  |  |  |  |  | 42.305 | 42.58 |
| Provean |  |  |  |  |  |  |  |  | 56.865 |
| Condel |  |  |  |  |  |  |  |  |  |

1. Glossary

Confidence score (C-score ) for estimating the quality of predicted models typically in the range of [-5,2]

Template Modeling Score (TM-score) measure the structural similarity between two structures TM-score >0.5 indicates a model of correct topology and a TM-score<0.17 means a random similarity.

Heavy atoms Root-Mean-Square Deviation (RMSD) with respect to the experimental structure.

number of structure decoys (Low temperature replicas) at an unit of space in the SPICKER cluster.
